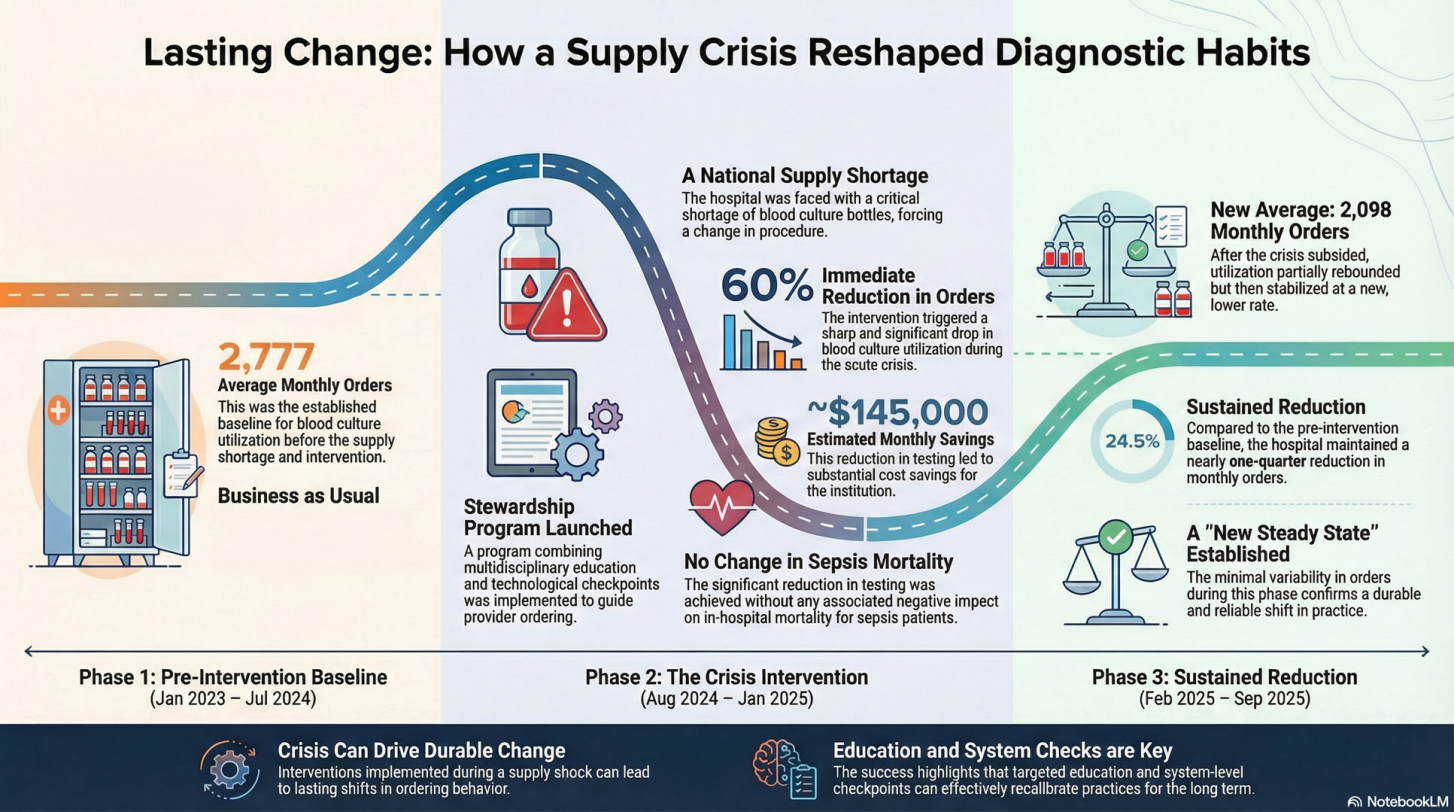# 186 Device Stewardship in Community Emergency Departments: Epidemiology of Unnecessary Indwelling Urinary Catheter Placement

**DOI:** 10.1017/ash.2026.10578

**Published:** 2026-06-23

**Authors:** Lior Cohen Yatziv, Scott Borgetti, Alfredo Mena Lora, Alan Gross, Nahed Ismail, Jenna Adams

**Affiliations:** 1 University of Illinois Chicago; 2 University of Illinios; 3 University of Illinois at Chicago; 4 Univ. of Illinois at Chicago College of Pharmacy; 5 UI Health

## Abstract

**Background:** Faced with a national blood culture (BC) bottle shortage, our institution implemented a diagnostic stewardship intervention relying on multidisciplinary teams' education to enhance provider awareness, along with technological checkpoints. This initial effort resulted in a 60% reduction in BC orders and an estimated monthly savings of approximately $145,000, with no change in in-hospital sepsis mortality. The initial report covered the post-intervention period up to January 2025. As bottle availability normalized, acute-phase restrictions ended, while key stewardship components were deliberately maintained. The electronic medical record (EMR) best practice advisory (BPA) remained active, paired with continued clinician education and ongoing monitoring of blood culture utilization to reinforce appropriate practice. We subsequently evaluated the durability of this stewardship approach, focusing on long-term utilization drift and the net sustained reduction in blood culture use after the acute supply crisis resolved. **Methods:** We conducted a retrospective review of BC utilization at a 438-bed tertiary care academic center. The study compared the Pre-Intervention Baseline (P1: January 2023-July 2024) against the two post-intervention phases: the acute shortage (P2: August 2024–January 2025) and the sustainability phase (P3: February 2025–September 2025). The stewardship intervention in P3 included an EMR-based BPA, along with ongoing education and monitoring. The primary outcome was the mean monthly BC orders. Statistical stability in P3 was assessed using 95% confidence intervals (CI). **Results:** Blood culture utilization averaged 2,777 sets per month during the pre-intervention period (P1). Following the acute shortage (P2), utilization partially rebounded and then stabilized at a lower plateau during the eight-month sustainability phase (P3) at 2,098 monthly orders. This represents a sustained 24.45% reduction compared with the pre-intervention baseline. The stability of P3 is supported by a narrow 95% confidence interval (1,909.92 to 2,186.08) and a low margin of error of ±88.08 orders, confirming that the reduction reflects a reliable new steady state. **Conclusion:** Our long-term evaluation shows that a diagnostic stewardship intervention implemented during an acute supply shock resulted in a durable shift in practice. Even after the shortage was resolved, monthly blood culture utilization remained nearly one-quarter below baseline and stabilized with minimal variability, indicating the establishment of a reliable new steady state. These findings highlight that targeted education and system-level checkpoints can drive lasting reductions in unnecessary testing and recalibrate ordering behavior well beyond the immediate crisis.

**Figure:**
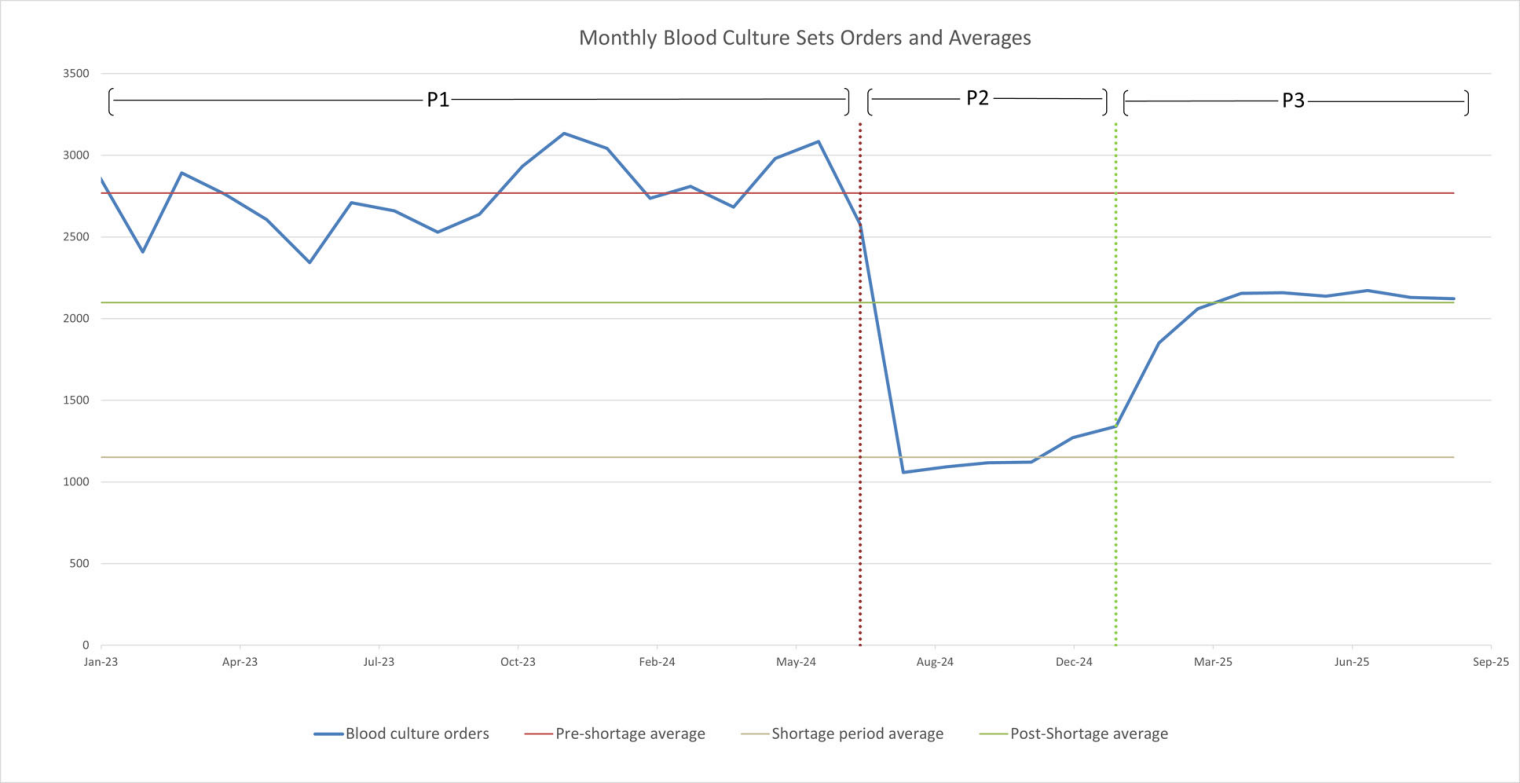

**Figure:**